# MRI Analysis of Paraspinal Muscle Morphology in Patients With Chronic Low Back Pain: A Case-Control Study

**DOI:** 10.7759/cureus.94072

**Published:** 2025-10-07

**Authors:** Nishanth Varma, Harini Bopaiah, Anil K Sakalecha, Rishi Prajwal H L, Sneha D N

**Affiliations:** 1 Department of Radiodiagnosis, Sri Devaraj Urs Medical College, Kolar, IND

**Keywords:** atrophy, chronic, degeneration, inflammation, muscle fat infiltration, muscles, pain, spine, vertebrae

## Abstract

Background: Chronic low back pain (CLBP) is a major contributor to musculoskeletal disability worldwide. The paraspinal muscles, particularly those along the thoracolumbar spine, are essential for spinal stability and extension. Morphological alterations in these muscles may have diagnostic and therapeutic implications. This study aimed to determine the cross-sectional area (CSA) of each paraspinal muscle at the level of the L4 superior endplate in patients with chronic back pain (CBP) compared to age-matched individuals without any history of low back pain. We hypothesized that patients with CLBP would demonstrate selective reductions in the CSA of the multifidus, while the psoas major (PS) would remain relatively preserved.

Methods: A cross-sectional observational study was conducted with 80 participants: 40 patients with CBP and 40 age-matched controls without low back pain. The PS, multifidus, erector spinae (ES), and quadratus lumborum (QL) muscles were assessed bilaterally using magnetic MRI. An independent t-test was used to assess the statistical significance between the two groups, with significance set at p < 0.05.

Results: The mean ages of the cases and controls were 50.54 and 49.94 years, respectively, with no statistically significant difference. Among the paraspinal muscles evaluated, statistically significant differences in the CSA were found in the multifidus, ES, and QL muscles, but not in the PS.

Conclusion: Statistically significant reductions in the CSA were observed in most paraspinal muscles, except for the PS, in patients with CBP. These preliminary, hypothesis-generating findings highlight the potential value of paraspinal muscle assessment in CLBP and warrant confirmation in larger, multi-center studies before therapeutic implications can be considered.

## Introduction

Low back pain (LBP) is a highly prevalent, serious, and expensive musculoskeletal health concern, deemed the leading cause of disability, impacting individuals of all ages, socioeconomic backgrounds, and geographies (especially working-aged people) [1-4]. Over the last decade, the overall incidence of LBP among adults has increased twofold, with the elderly experiencing the greatest increase. The global incidence of activity-limiting LBP remained at 7.3% in 2015, affecting over 500 million people [1,3,4]. LBP is described as pain or muscular stiffness occurring between the posterior costal margin and the inferior gluteal folds, regardless of origin. Chronic back pain (CBP) refers to LBP of moderate-to-severe intensity (on the Oswestry Disability Index used in our study) [5]. By 2050, CBP is expected to be the root cause of impairment in 36.4% of the global population. Based on the existing epidemiological evidence, the frequency, prevalence, and handicap caused by CBP are practically the same worldwide, independent of geography, at 3.2%, 7.6%, and 2.6%, respectively [5]. It is a multi-factorial disease, wherein patients with CBP usually have moderate-to-severe paraspinal muscular atrophy, which manifests as a diminished cross-sectional area (CSA) of the paraspinal muscles. LBP differs according to the duration and frequency of pain [5]. Scholars have long recognized the relationship between chronic LBP and structural changes in paravertebral muscles [1]. The paraspinal muscles located in the thoracic and lumbar spine work together to stretch the spine, apart from stabilizing the spine [6]. Increased muscular activity helps stabilize intervertebral motion segments throughout bending and other spinal movements. They actively engage in motions that involve flexion, extension, lateral flexion, and rotation [6]. Intervertebral disc problems are common imaging findings in patients with CBP. However, studies have shown that such situations do not contribute to the CBP [5,7]. The multifidus and erector spinae (ES) are important core muscles that work together to support the spine while controlling movement [8,9]. Atrophy of these muscles can lead to LBP, which differs according to the duration and frequency of pain [10]. Whenever imaging beyond simple radiographs is clinically necessary, cross-sectional MRI is commonly employed to diagnose the cause of LBP [3].

The present study aimed to evaluate paraspinal muscle morphology in patients with chronic LBP using MRI, focusing on potential selective atrophy patterns. We hypothesized that the multifidus would show a significant reduction in CSA compared with controls, while the psoas major (PS) would remain relatively preserved. Measurements were standardized at the L4 superior endplate, a reliable anatomical reference used in prior morphometric studies. By restricting the cohort to males aged 40-60 years, we minimized confounding from sex-based differences in muscle mass and age-related sarcopenia, thereby enhancing internal validity.

## Materials and methods

A cross-sectional observational study was designed and carried out among 80 subjects in the Department of Radiodiagnosis of Sri Devaraj Urs Medical College, Tamaka, Kolar, India. Our study design was approved by the ethics board, and all necessary approvals were obtained prior to the start of the study.

All participants were briefed about the study objectives and potential benefits in their native language, and informed consent was obtained prior to inclusion. A total of 80 subjects were enrolled and divided into two groups: Group A comprised 40 individuals with CBP, and Group B included 40 age-matched controls with no history of CBP.

The study included male patients aged 40-60 years who experienced moderate-to-severe CBP of moderate-to-severe intensity, as assessed using the Oswestry Disability Index. Additionally, age-matched male individuals who were referred for MRI of the abdomen/pelvis with no history of low back pain were included in the control group. Finally, participants who provided consent to undergo lumbar spine MRI as part of the study.

The study excluded female patients and individuals with a history of surgery involving the vertebral column or hip. Patients with a history of vertebral column or pelvic bone disorders were also excluded. Furthermore, obese patients with neurological disorders who had received treatment for back pain within the past six months were not eligible for participation. Finally, male patients who were either younger than 40 years or older than 60 years were excluded from the study.

We recorded a detailed, comprehensive medical history, followed by rating the intensity of chronic low back pain (CLBP) using the Oswestry Disability Index. All consented patients then underwent MRI using a 1.5 Tesla Siemens Magnetom Avanto MRI scanner.

**Figure 1:**
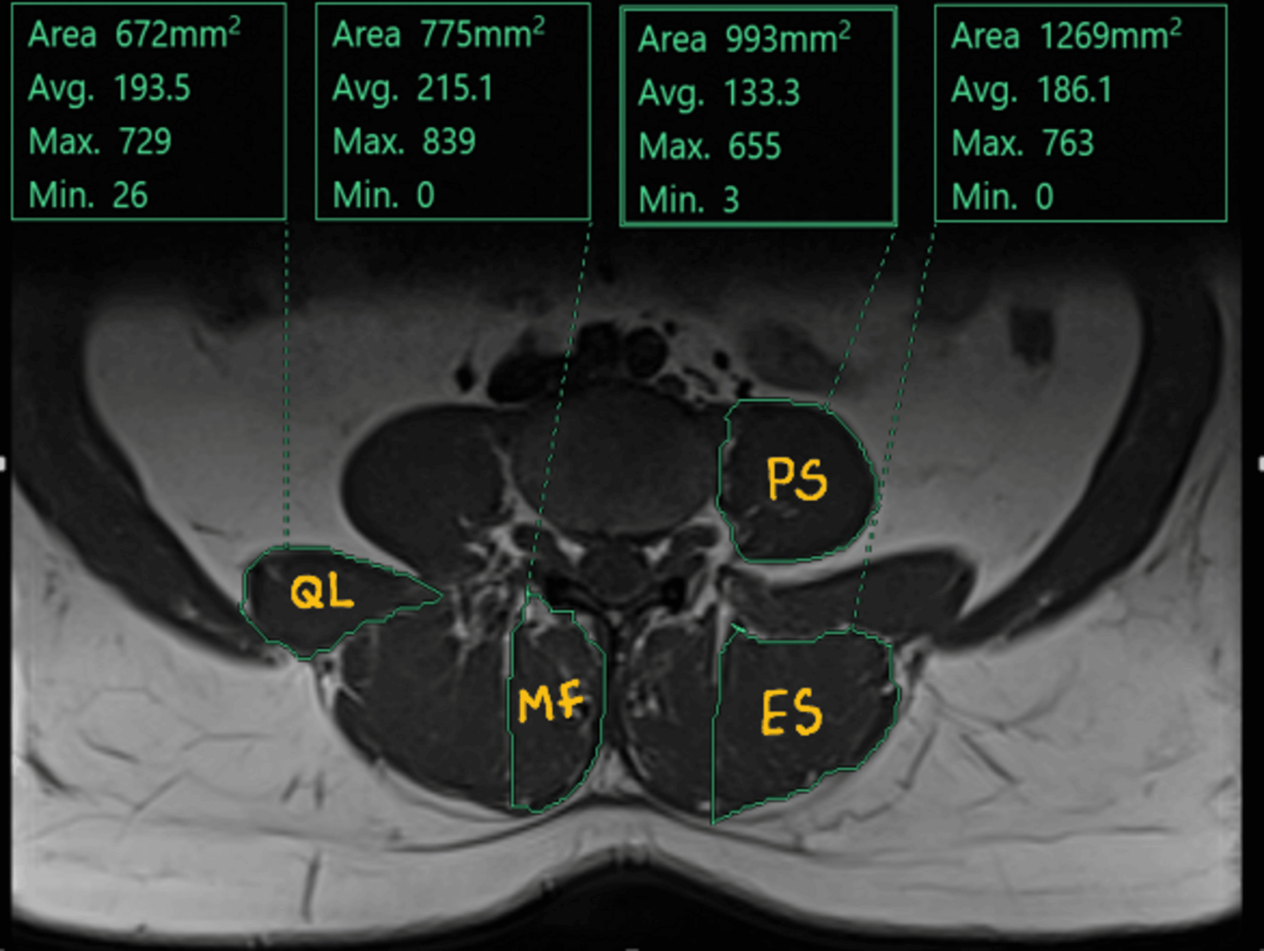
MRI axial T2-weighted image demonstrating the cross-sectional area (cm²) of paraspinal muscles—erector spinae, multifidus, psoas major, and quadratus lumborum—at the level of the L4 superior endplate. MRI: Magnetic Resonance Imaging; L4: Fourth Lumbar Vertebra; ES: Erector Spinae; MF: Multifidus; PS: Psoas Major; QL: Quadratus Lumborum

**Figure 2:**
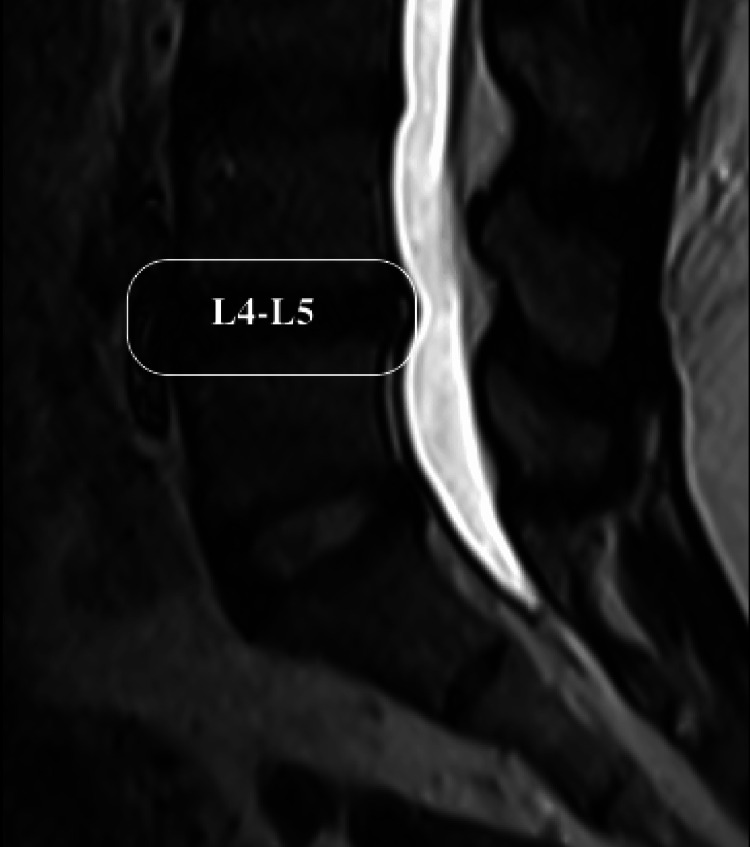
MRI sagittal T2W image of the lumbar spine demonstrating the L4-L5 intervertebral disc. MRI: Magnetic Resonance Imaging

MRI Imaging Protocol: The CSA of each paraspinal muscle was measured at the level of the L4 superior endplate in patients with CBP and in age-matched individuals with no history of CBP (Figure 1). Additionally, sagittal T2-weighted MRI images at the L4-L5 level were evaluated to identify intervertebral disc (IVD) abnormalities, such as standard alignment, bulge, protrusion, or extrusion, in patients with CBP (Figure 2).

CSA measurements were performed using ImageJ software (NIH, Bethesda, MD). Inter- and intra-observer reliability were assessed, with ICC values >0.90, confirming measurement consistency.

All images were independently reviewed by two blinded radiologists. Segmentation of muscle boundaries was performed manually on axial T2-weighted images at the L4 superior endplate using standardized anatomical landmarks. Inter-observer reliability was assessed on a random 20% subsample, with ICC values exceeding 0.90 across all muscles.

Sample size estimation: The sample size was calculated using the following formula:

N = (2 × SD² × (Zα/2 + Zβ) ²) / d²

In this formula, Zα/2 represents the critical value of the normal distribution at α/2 (e.g., for a 95% confidence level, α = 0.05, and Zα/2 = 1.96). Zβ is the critical value corresponding to the desired statistical power (for a power of 80%, β = 0.2, and Zβ = 0.84). SD is the standard deviation derived from the variance of a previously studied population, and d denotes the minimum expected difference between the two group means.

Statistical analysis: Data were entered into a Microsoft Excel () datasheet and analyzed using Statistical Product and Service Solutions (SPSS, version 22; IBM SPSS Statistics for Windows, Armonk, NY) software. Continuous data are represented as mean and standard deviation. An independent t-test was used to test the significance of the mean difference between the two groups, with a p-value <0.05 considered statistically significant.

Sample size: The sample size was estimated using the difference in the mean muscle fat ratio (MFR) between the cases and controls obtained from a pilot study, which reported values of 3.90 ± 0.65 for the cases and 4.56 ± 0.73 for the controls. Based on these values, with a 95% confidence level and 80% statistical power, a sample size of 38 subjects per group was calculated using the formula mentioned above and MedCalc sample size software. Accounting for a 10% non-response rate, the sample size was adjusted to approximately 40 participants per group.

## Results

The mean age of the cases and controls was 50.54 years and 49.94 years, respectively, which was found to be statistically insignificant. The mean CSA of the multifidus right (MFR) muscle in cases and controls was 3.82 cm^2^ and 4.86 cm^2^, respectively, and the mean CSA of the multifidus left (MFL) muscle was 3.89 cm² and 4.61 cm², respectively. These differences were found to be statistically significant (p < 0.05) (Table 1).

**Table 1:** Comparison of the mean, standard deviation, and median cross-sectional area (CSA) of right and left multifidus muscles (MFR and MFL) between cases and controls. S.D: Standard Deviation, CSA: Cross Sectional Area, QLR: Quadratus Lumborum Right, QLL: Quadratus Lumborum Left Pearson's chi-squared test; p<0.001: highly significant; p<0.05: significant

Muscle group	Group	Mean ± SD (cm²)	Range (cm²)	Median (cm²)	t-value	p-value
MFR CSA	Cases	3.82 ± 0.31	3.4-4.4	3.8	–10.28	<0.001
	Controls	4.86 ± 0.56	4.0-5.6	4.9		
MFL CSA	Cases	3.89 ± 0.33	3.0-4.4	4	–7.39	<0.001
	Controls	4.61 ± 0.52	3.8-5.5	4.7		

The mean CSA of the ES right (ESR) muscle in cases and controls was 13.0 cm^2^ and 12.1 cm^2^, respectively, and the mean CSA of the ES left (ESL) muscle was 13.30 cm^2^ and 12.1 cm^2^, respectively. These differences were found to be statistically significant (Table 2).

**Table 2:** Comparison of the mean, standard deviation, and median cross-sectional area (CSA) of the right and left erector spinae muscles (ESR and ESL) between cases and controls. S.D: Standard Deviation, CSA: Cross Sectional Area, ESR: Erector Spinae Right, ESL: Erector Spinae Left Pearson's chi-squared test; p<0.001: highly significant; p<0.05: significant

Muscle group	Group	Mean ± SD (cm²)	Range (cm²)	Median (cm²)	t-value	p-value
ESR CSA	Cases	13.0 ± 1.79	10.5-16.1	13	2.55	0.012
	Controls	12.1 ± 1.33	9.4-13.5	12.4		
ESL CSA	Cases	13.3 ± 1.82	10.5-16.2	12.9	2.72	0.008
	Controls	12.1 ± 2.12	9.0-14.0	11.9		

The mean CSA of the psoas right (PSR) muscle in cases and controls was 7.65 cm^2^ and 7.88 cm^2^, respectively, and the mean CSA of the psoas left (PSL) muscle was 7.63 cm² and 7.67 cm², respectively. These differences were found to be statistically insignificant (Table 3).

**Table 3:** Comparison of the mean, standard deviation, and median cross-sectional area (CSA) of the right and left psoas muscles (PSR and PSL) between cases and controls. S.D: Standard Deviation, CSA: Cross Sectional Area, PSR: Psoas Major Right, PSL: Psoas Major Left Pearson's chi-squared test; p<0.001: highly significant; p<0.05: significant

Muscle group	Group	Mean ± SD (cm²)	Range (cm²)	Median (cm²)	t-value	p-value
PSR CSA	Cases	7.65 ± 1.17	6.1-9.4	7.5	-0.82	0.22
	Controls	7.88 ± 1.33	6.1-9.9	7.5		
PSL CSA	Cases	7.63 ± 1.01	6.5-9.2	7.2	-0.18	0.49
	Controls	7.67 ± 1.01	6.5-9.1	7.2		

The mean CSA of the QL right (QLR) muscle in cases and controls was 5.31 cm^2^ and 4.86 cm^2^, respectively, and the mean CSA of the QL left (QLL) muscle was 5.69 cm^2^ and 5.28 cm^2^, respectively. These differences were found to be statistically significant (Table 4).

**Table 4:** Comparison of the mean, standard deviation, and median cross-sectional area (CSA) of the right and left quadratus lumborum muscles (QLR and QLL) between cases and controls. S.D: Standard Deviation, CSA: Cross-Sectional Area, QLR: Quadratus Lumborum Right, QLL: Quadratus Lumborum Left Pearson's chi-squared test; p<0.001: highly significant; p<0.05: significant

Muscle group	Group	Mean ± SD (cm²)	Range (cm²)	Median (cm²)	t-value	p-value
QLR CSA	Cases	5.31 ± 1.07	3.7-6.5	5.9	2.13	0.035
	Controls	4.86 ± 0.80	3.5-6.1	4.8		
QLL CSA	Cases	5.69 ± 0.99	4.0-6.9	6.1	1.97	0.052
	Controls	5.28 ± 0.87	3.7-6.5	5.5		

In the CLBP cohort (n = 40), qualitative MRI analysis at the L4-L5 level revealed that 14 patients (35%) exhibited disc abnormalities such as bulge, protrusion, or extrusion on sagittal T2-weighted images. To evaluate the potential impact of these disc abnormalities on paraspinal muscle morphology, the CSA values of the relevant muscles were compared between patients with and without disc lesions. However, no statistically significant differences were observed, consistent with findings reported in previous studies.

## Discussion

The multifidus muscle plays a critical role in segmental regulation and stabilization of the lumbar spine. It contributes significantly to the neuromuscular control of the lower trunk through feedback mechanisms mediated by proprioceptors densely packed within muscle spindles. In patients with CBP, reduced trunk muscle CSA is commonly observed, particularly at the L4-L5 level, where site-specific muscle alterations have been documented [5]. The multifidus alone is responsible for maintaining approximately two-thirds of the active lumbar spinal stability, with the remaining support provided by the PS, erector spinae, and QL muscles. MRI remains the gold standard for evaluating paraspinal muscle morphology in CBP, as it enables precise delineation of individual muscles, accurate measurement of the CSA, and differentiation of muscle tissue from fat, facilitating the calculation of the muscle-to-fat ratio [5].

MRI is preferred over computed tomography (CT) for the evaluation of spinal structures because of its superior soft tissue contrast and radiation-free imaging capabilities. It enables high-resolution cross-sectional imaging of various spinal components, including the intervertebral discs, nerve roots, facet joints, and paraspinal musculature (PSM). Although many MRI-detected abnormalities appear with similar prevalence in individuals with and without LBP, certain features, such as vertebral endplate lesions, disc bulges, herniations, and spondylolysis, are more commonly observed in symptomatic patients [3].

In our study, the mean ages of the case and control groups were 50.54 and 49.94 years, respectively, a difference that was statistically insignificant. Liu et al. [10] reported comparable findings, with mean ages of 41.43 years in the cases and 40.82 years in the controls. In contrast, Gupta et al. documented a mean ± SD age of 39.17 ± 12.82 years in their cohort; however, their study did not include a control group for comparison [11].

The multifidus muscle in the lumbar region plays a key role in maintaining the normal curvature of the spine by exerting targeted segmental control over the individual lumbar vertebrae. Segmental fibers of the multifidus have been shown to influence the spine’s ability to bear axial loads by regulating vertebral rotation and facilitating the distribution of mechanical stress to adjacent anatomical structures [5,11,12]. These muscles are also integral to the neuromuscular control of the lumbar spine, providing critical feedback through proprioceptors located within their densely packed muscle spindles [5]. Such feedback mechanisms are essential for coordinating both the static posture and dynamic movements of the musculoskeletal system. Importantly, significant atrophy of the deep fibers of the multifidus has been associated with a higher likelihood of experiencing severe back pain than atrophy in other paraspinal muscles [5].

Multifidus atrophy is most frequently observed in the lower lumbar spine, particularly at the L4 and L5 levels in individuals with LBP, with reported prevalence rates of 13.5% and 12.2%, respectively [5,13]. In patients with CBP, the CSA of the multifidus muscle at these levels typically ranges from 3.47 to 7.08 cm^2^, compared to 4.61-7.65 cm^2^ in healthy individuals.

In our study, the mean CSA of the right multifidus (MFR) was 3.82 cm^2^ in the cases and 4.86 cm^2^ in the controls, while the mean CSA of the left multifidus (MFL) was 3.89 cm^2^ and 4.61 cm^2^, respectively. These differences were statistically significant, indicating a consistent pattern of multifidus atrophy in patients with CBP.

Comparable findings were reported by Mamatha et al., who documented median MFR CSA values of 3.94 cm^2^ in cases and 4.56 cm^2^ in controls, and median MFL values of 3.97 cm^2^ and 4.52 cm^2^, respectively [5]. In contrast, Gupta et al. reported higher multifidus CSA values, with a mean ± SD MFR of 5.74 ± 1.52 cm^2^ and a median of 6.50 cm^2^ (range: 4.83-6.91); however, their study lacked a control group for comparison [11]. A recent meta-analysis discovered atrophy and increased fatty infiltration of the lumbar multifidus (LM) muscle in those with LBP, while the ES and PS did not show significant abnormalities [2,14]. However, contradictions persist in the literature. While many studies have reported atrophy of the LM, others have found no significant change or even hypertrophy, along with increased LM fat infiltration [2].

In our cohort, the observed reduction in multifidus size likely reflects chronic disuse and denervation changes commonly associated with prolonged LBP.

A recent study found that the multifidus of patients with CBP showed selective atrophy, whereas the ES showed modest compensatory hypertrophy [14].

The ES is another muscle that helps maintain the posture and stability of the spinal column. Similar to the multifidus, they have a more sluggish twitch and greater size [5].

The mean ESR of cases and controls in our study was 13.0 cm^2^ and 12.1 cm^2^, respectively, and the mean ESL was 13.30 cm^2^ and 12.1 cm^2^, respectively, which was found to be statistically significant.

Hyperactivity of the ES muscles in individuals with back pain has been linked to increased muscular control and spinal stability as protective mechanisms [5]. In our CBP cohort, the ES CSA was notably larger, which may reflect differences in patient selection, such as community-dwelling CBP subjects versus those recovering from acute pain or variation in the anatomical levels measured. This enlargement may result from chronic muscle guarding or prolonged eccentric loading. Chronic pain is known to alter neuromuscular control, and some studies have suggested that the lumbar extensors become overactive to compensate for spinal instability. Our findings are consistent with those of Schönnagel et al., who also reported increased paraspinal CSA in patients with CBP.

However, other studies have reported slightly lower ES muscle CSA values. Mamatha et al. documented median ESR values of 12.96 cm^2^ in cases and 10.62 cm^2^ in controls, while median ESL values were 13.42 cm^2^ and 10.55 cm^2^, respectively [5]. Similarly, Gupta et al. reported a mean ± SD ESR CSA of 12.89 ± 3.05 cm^2^, with a median of 12.44 cm^2^ (range: 14.43-20.68) [10].

The PS is an important muscle to examine in individuals with CBP because it strengthens the lumbar spine from L1 to L5. Strengthening the lumbar area of the spine is essential for pelvic stability [15]. Studies have examined the relationship between CSA of the PS muscle and symptomatic lumbar spinal stenosis and discovered that the PS has no change in muscle mass and seldom experiences fatty infiltration [5]. Thus, discomfort in these patients may be attributable to nerve compression rather than muscle morphometric alterations. The mean CSA of the PSR in the cases and controls of our study was 7.65 cm^2^ and 7.88 cm^2^, respectively, and the mean PSL was 7.63 and 7.637 cm^2^, respectively, which was found to be statistically insignificant. In comparison to the left side, we found the right side to be higher, which was the reverse of that observed in the case of the other muscles. Mamatha et al. reported median PSR in cases and controls was 7.40 cm^2^ and 7.28 cm^2^, and median PSL was 7.80 and 7.09 cm^2^, respectively [5], which was in agreement with the results of our study. The PS was unaffected in our cases, which is consistent with several studies. Lee et al. [13] found no significant psoas CSA difference at L5 in chronic patients, and others have shown that the psoas often retains volume even in symptomatic spine disorders. The psoas is a powerful hip flexor rather than a primary spinal stabilizer; its anatomy and innervation may spare it from the reflex atrophy seen in the multifidus. From a clinical standpoint, this suggests that psoas MRI morphometry may be less useful for CLBP assessment than that of the paraspinal core muscles. The QL is the deepest muscle of the posterior abdominal wall and connects the lumbar vertebrae to the pelvis [5]. It supports the lumbar spine in the erect position, in addition to the lateral direction when bending. This muscle has several fascicles and is recognized as a trigger point for low backache whenever it is overworked, pressured, or strained [5]. The mean CSA of the QLR in our cases and controls was 5.21 cm^2^ and 4.89 cm^2^, respectively, and the mean QLL was 5.59 cm^2^ and 5.28 cm^2^, respectively, which was found to be statistically significant.

Mamatha et al. reported median CSA values of the QLR as 4.58 cm^2 ^in cases and 4.43 cm^2^ in controls, and median CSA values of the QLL as 5.16 cm^2^ and 4.83 cm^2^, respectively. These measurements were comparatively lower than those observed in the present study [5].

The QL muscle exhibited modest hypertrophy in our CLBP cohort. As a deep lateral stabilizer of the spine, the QL may be subject to chronic activation due to compensatory postural adjustments in the setting of persistent LBP, potentially leading to an increase in its CSA [16]. Although few quantitative imaging studies have evaluated QL morphology in LBP, it is frequently cited in the literature as a potential pain generator or an “overworked” muscle. Our finding of an enlarged QL CSA may reflect muscular overuse or sustained hypertonicity. Further research is required to clarify the clinical significance of this observation. Notably, a recent observational study linked QL stiffness with pain characteristics, highlighting the muscle’s emerging relevance in CLBP assessment, although that study assessed stiffness rather than morphometric changes [10].

In this study, > one-third of the patients exhibited L4-L5 disc abnormalities on MRI, with a prevalence of approximately 35%. This finding aligns with previous imaging studies, as meta-analytic data indicate that lumbar disc bulges are present in approximately 40-45% of symptomatic adults, compared to only ~6% in asymptomatic individuals. However, it is important to note that the presence of disc lesions in CLBP does not always imply clinical significance. Numerous asymptomatic adults demonstrate disc bulges on MRI, underscoring the necessity of careful clinical correlation when interpreting the imaging findings. Although disc abnormalities were observed in one-third of patients, these did not significantly alter muscle CSA values. This suggests that muscle morphology changes occur independently of disc pathology, consistent with prior reports, though larger studies are needed to explore these relationships in depth.

When present, L4-L5 disc herniations may exacerbate spinal instability or nerve irritation, contributing to chronic pain and reduced physical activity. This can initiate a cascade of musculoskeletal deterioration, consistent with the proposed “vicious cycle” of CLBP, wherein pain leads to disuse, resulting in paraspinal muscle deconditioning, fat infiltration, and atrophy, which in turn perpetuates or worsens the pain experience [17,18].

Conversely, primary paraspinal muscle weakness or fatty infiltration may compromise spinal support, potentially accelerating intervertebral disc degeneration (IVDD). The observed association between IVDD severity and muscle changes suggests a bidirectional relationship in which muscular and discal pathologies may perpetuate each other.

Given their essential role in stabilizing the thoracic and lumbar spine, any structural or functional impairment of the paraspinal muscles can significantly affect spinal biomechanics and the overall quality of life. Disorders affecting these regions often lead to pain, stiffness, and reduced mobility in the neck. Therefore, early identification and appropriate management of such conditions are vital for preserving spinal function and patient well-being [6]. Given the cross-sectional nature of our design, causal inferences cannot be drawn, and our findings should be interpreted as associations rather than directional effects.

Limitations: Although we assessed all parameters required for our study, several shortcomings must be acknowledged. The limited sample size and single-center, non-randomized design restrict the statistical power and generalizability of our findings. Restricting enrollment to males aged 40-60 years helped minimize variability from sex-related muscle differences and age-related sarcopenia, but considerably narrows external validity and prevents extrapolation to broader populations. Consequently, our results should be regarded as exploratory and hypothesis-generating rather than definitive. In addition, we did not quantify intramuscular fat infiltration, an important marker of muscle quality, nor did we perform formal intra- and inter-rater reliability testing of CSA measurements. These omissions represent key methodological limitations that future studies should address through larger, multicenter, randomized, and multi-observer designs, including both sexes and wider age ranges.

## Conclusions

All spinal muscles contribute to stabilization; however, consistent with prior research, our study found that the multifidus and ES showed the most notable alterations in patients with CLBP, while the PS was relatively preserved. These results are broadly consistent with the concept of chronic muscle degeneration and compensatory hypertrophy. At the same time, our small, selective, cross-sectional cohort limits generalizability, and the findings should be considered preliminary and hypothesis-generating rather than definitive. While these associations may eventually inform targeted rehabilitation strategies, any therapeutic implications remain speculative until validated in larger, multi-center, longitudinal studies incorporating more diverse populations and comprehensive muscle quality metrics.
